# A novel reconstruction technique of a tracheal defect in the emergent setting using a thymus flap in a patient with tracheoinnominate artery fistula

**DOI:** 10.1186/s40792-019-0763-x

**Published:** 2020-01-14

**Authors:** Tyler B. Draeger, Shahriyour K. Andaz, Vanessa R. Gibson

**Affiliations:** 1 Department of Surgery, Icahn School of Medicine at Mount Sinai South Nassau, Oceanside, NY USA; 2 Division of Cardiothoracic Surgery, Department of Surgery, Icahn School of Medicine at Mount Sinai South Nassau, Oceanside, NY USA

**Keywords:** Tracheoinnominate fistula, Tracheal bleeding, Pedicled flap, Tracheal reconstruction, Thymus

## Abstract

**Background:**

There is a very high mortality associated with a tracheoinnominate artery fistula; however, when patients survive, they often require reconstruction of the eroded tracheal defect after the bleeding has been controlled.

**Case presentation:**

This is the case of an 83-year-old male with a tracheoinnominate artery fistula who was stabilized in the operating room and underwent repair of his trachea. A novel technique of using the thymus gland as a pedicled flap to repair a large tracheal defect was executed after achieving hemostasis. The patient’s defect was repaired successfully following control of the fistula.

**Conclusions:**

We have shown that the thymus gland can be used successfully as a pedicled flap for repair of a tracheal defect in the setting of a tracheoinnominate artery fistula.

## Introduction

The tracheoinnominate artery fistula is one of the most feared complications patients experience after creation of a tracheostomy for long-term mechanical ventilation. This is because patients who develop a tracheoinnominate artery fistula seldom survive due to exsanguination through the fistula. The mortality of a tracheoinnominate artery fistula is estimated to be 88% within the first 2 months after a fistula with the majority of patients dying during the initial attempt at hemorrhage control [1]. The treatment involves placing a gloved finger into the tracheostomy site or overinflating the cuff of the tracheostomy tube and rushing the patient to surgery to control the hemorrhage and repair the eroded trachea, if the patient survives this long.

The etiology of the fistula is thought to be multifactorial. Some of the causes include malpositioning of a cannula tip resulting in mucosal trauma, a low tracheal incision, excessive neck movement or extension, radiation, high cuff pressures, and prolonged intubation [2]. Other risk factors include infection, glucocorticoid use, hypotensive episodes, and malnutrition [3].

Once the patient is initially stabilized, reconstruction of the trachea is necessary. Scattered case reports identify using primary closure with simple sutures to close the defect, a bovine pericardial patch, or muscle flaps such as the pectoralis major muscle [3]. In more controlled situations, such as planned resections of the trachea, alternative reconstruction techniques are also described [4, 5]. Some authors also recommend reconstruction of the brachiocephalic trunk to manage stroke in cases of poor collateralization in the Circle of Willis; however, the rate of lethal rebleeding has been observed to be increased when this is performed. The rate of neurological deficit is reported to be approximately 10% in patients with innominate artery ligation [3]. In fact, some authors have reported elective ligation of the tracheoinnominate artery as a prophylactic measure to prevent tracheoinnominate artery fistula [6].

## Case presentation

This is the case of an 83-year-old male who presented to the hospital initially with low-grade fever and was subsequently identified to have *Clostridium difficile* colitis. The patient was admitted from a skilled nursing facility for ventilator-dependent patients with a tracheostomy tube in situ. His history was significant for cerebral vascular accident (CVA) 3 times, atrial fibrillation (AF), abdominal aortic aneurism (AAA), chronic obstructive pulmonary disease (COPD), neurogenic dysphagia (for which he had a percutaneous endoscopic gastrostomy tube placement (PEG)), stage 4 sacral decubitus ulcer with osteomyelitis, type 2 diabetes, hypertension, chronic renal failure, peripheral vascular disease, previous gastrointestinal bleeding, anemia, dementia (minimally responsive at baseline), a seizure disorder, and ventilator-dependent respiratory failure for which he had a tracheostomy created. The patient had a prolonged hospital stay, and on hospital day 33, he was noted to have bright red blood draining profusely from his tracheostomy tube. The patient underwent immediate evaluation by the cardiothoracic surgery team. The patient was noted to be losing blood and bradycardic. He went into cardiac arrest and pulseless electrical activity (PEA), but regained return of spontaneous circulation (ROSC) after 8 min of compression and massive transfusion. The patient was intubated orally and he was emergently taken to the operating room while the site was compressed with a gloved hand to tamponade the bleeding. Upon arrival at the operating room, the patient sustained another episode of cardiac arrest and chest compressions were resumed until ROSC was again achieved.

**Fig. 1:**
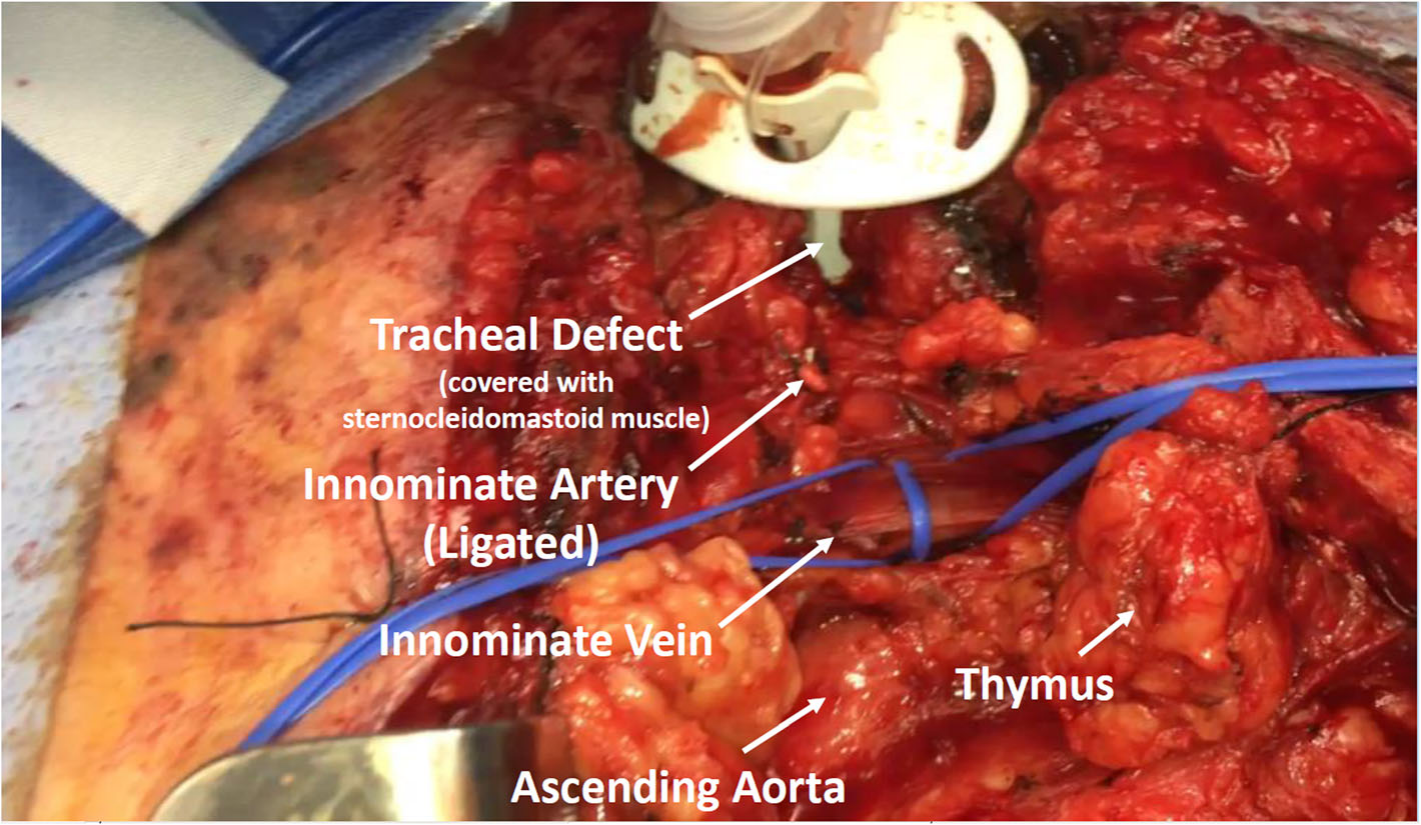
Identification of anatomy with partial closure with the sternocleidomastoid muscle

A median sternotomy incision was made from the tracheostomy stoma site down to the xiphoid process and the sternum opened with a sternal saw. Immediate packing was performed. There was an approximately 1-cm hole in the innominate which was immediately ligated with a 3-0 Prolene suture. This controlled the hemorrhage. A systematic inspection of the remaining portion of the chest cavity for hemostatic purposes was then performed as the patient was receiving massive transfusion protocol and developed significant coagulopathy. Intraoperative bronchoscopy was performed that showed inspissated blood within the airways, but no ongoing bleeding was identified. The anterior wall of the trachea was completely gone, with a significant erosion in this area, and it was determined that the patient would require a significant reconstruction. Chest tubes were placed and a povidone-iodine-soaked cling was used to pack with an overlying temporary closure with an Esmarch patch applied to close the chest. The patient was then transferred to the critical care unit for critical care management with an intent to come back when the patient was more stable for further management. The next day, the patient was taken back to the operating room. After removal of the temporary dressing, meticulous inspection revealed a significant anterior wall defect of the trachea with most of the cartilaginous rings extending from approximately ring 2 through ring 5 missing. The endotracheal tube was removed, and a temporary tracheostomy tube was placed using a #8 Shiley tracheostomy tube through the defect in the trachea. Inspection of the innominate artery and brachiocephalic vein identified that the vein was intact with no evidence of bleeding or direct communication with the tracheal wall defect and the artery’s ligating sutures remained intact with no evidence of bleeding (Fig. 1).

**Fig. 2:**
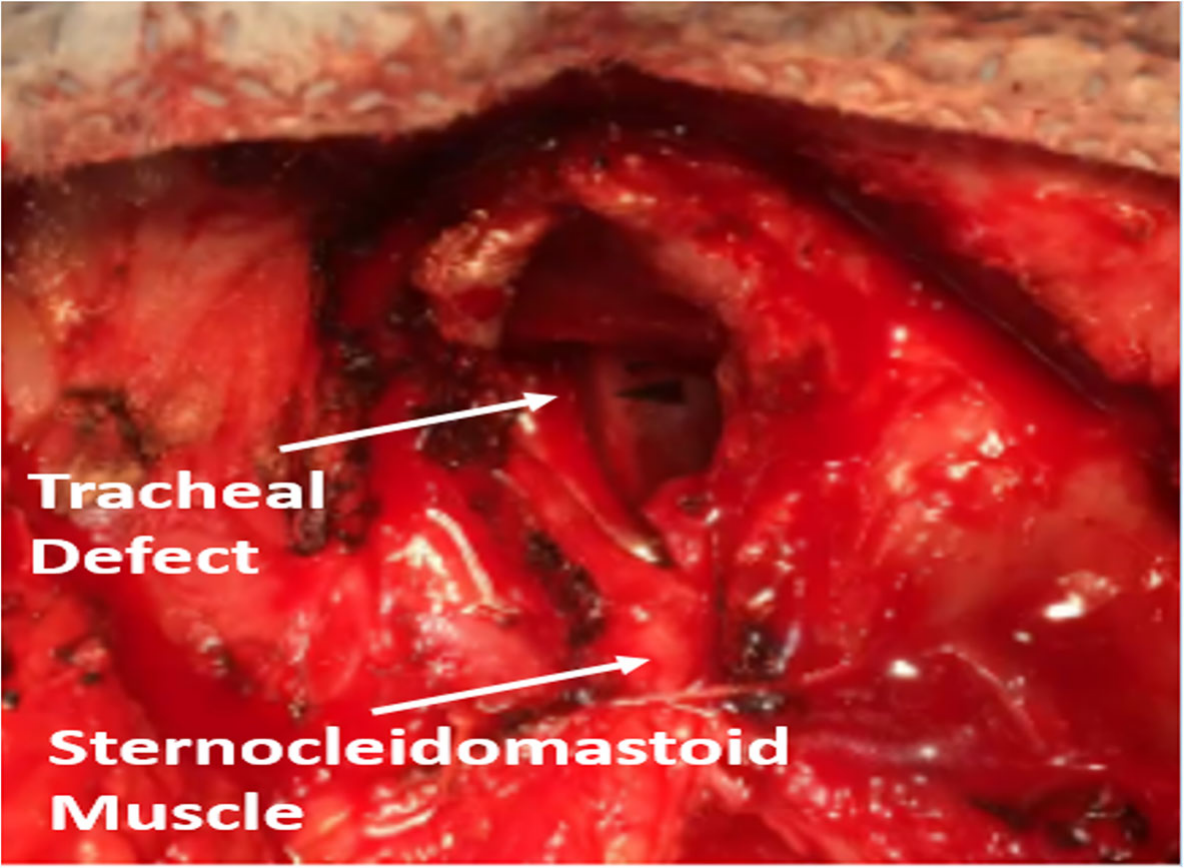
Identification of the large tracheal defect

**Fig. 3:**
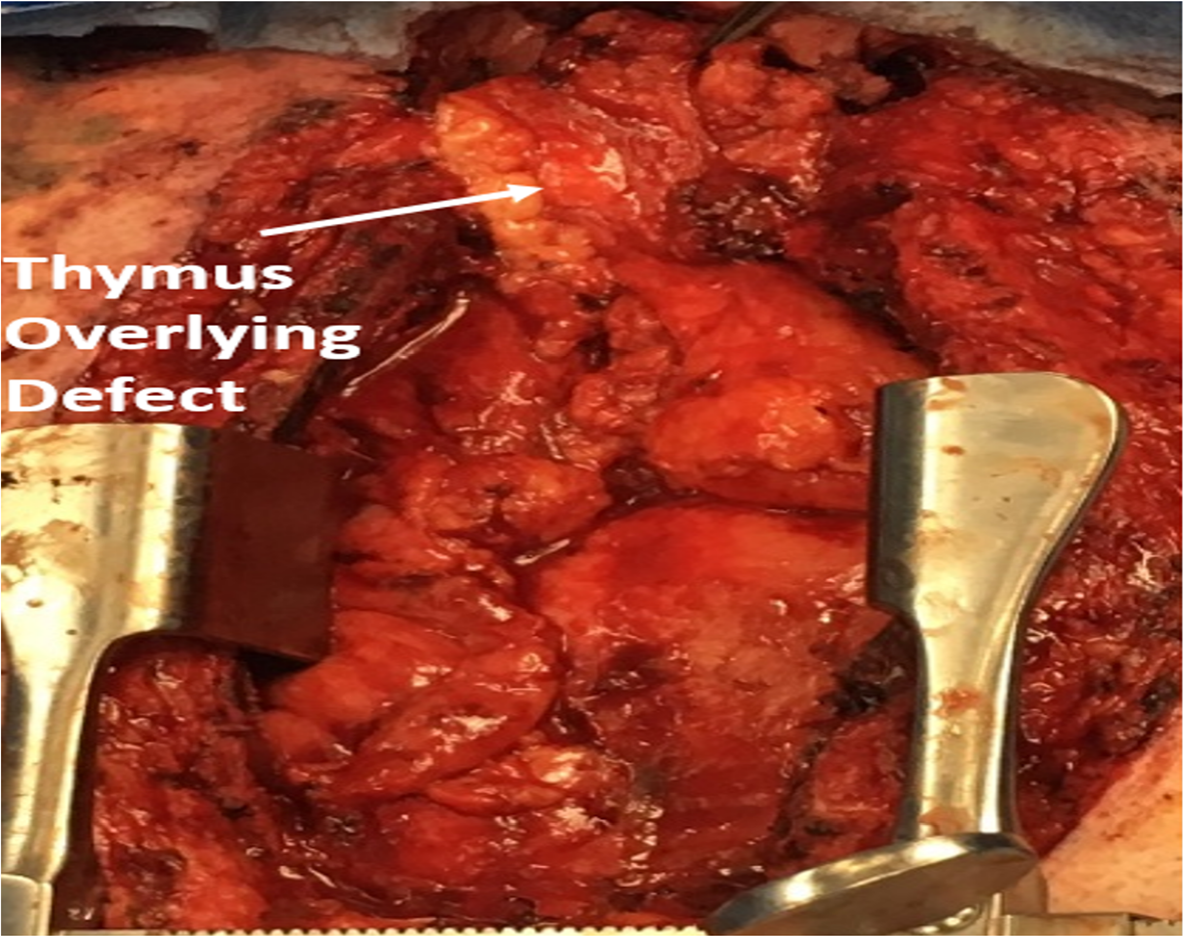
Thymus mobilized to cover tracheal defect

**Fig. 4:**
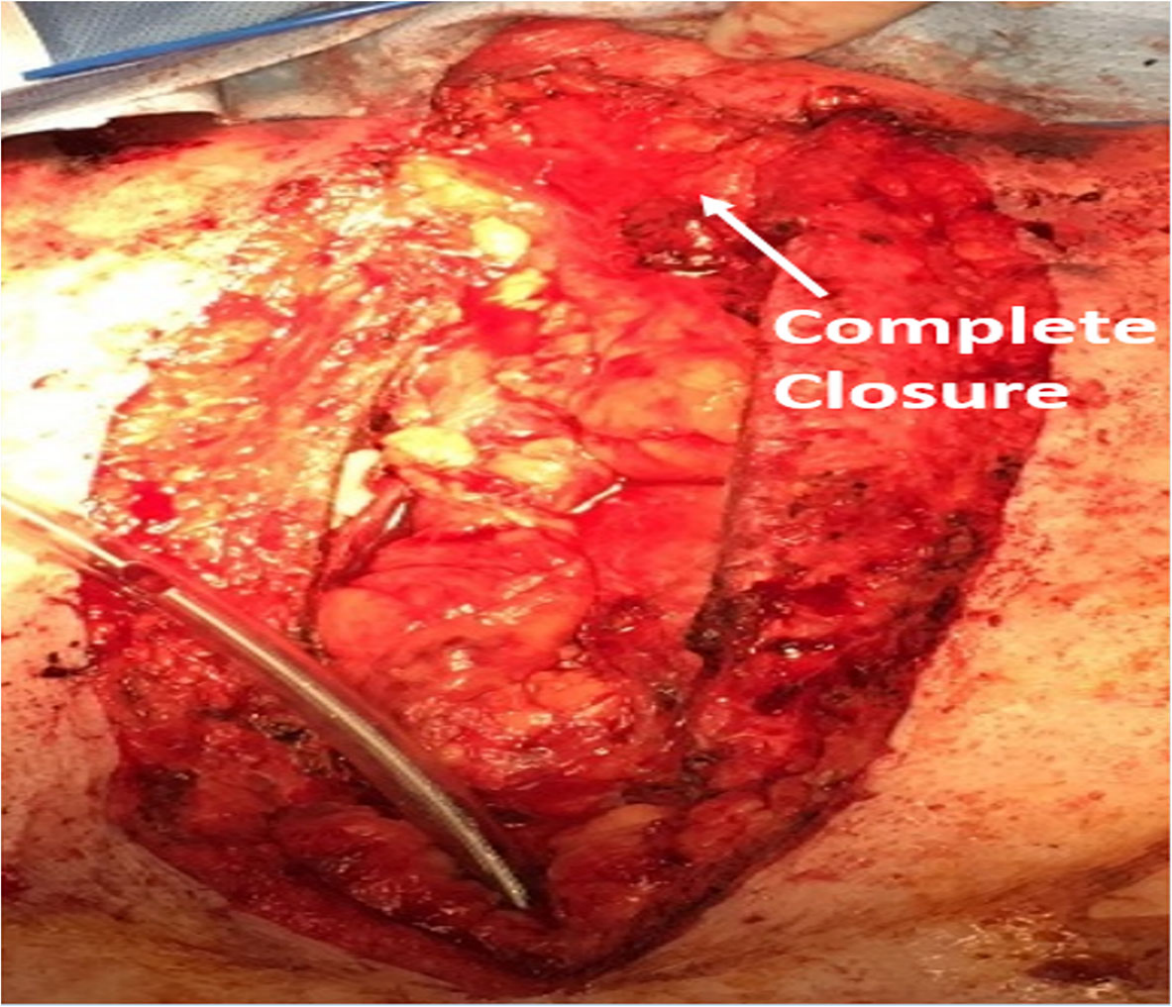
Complete closure of tracheal defect

**Fig. 5:**
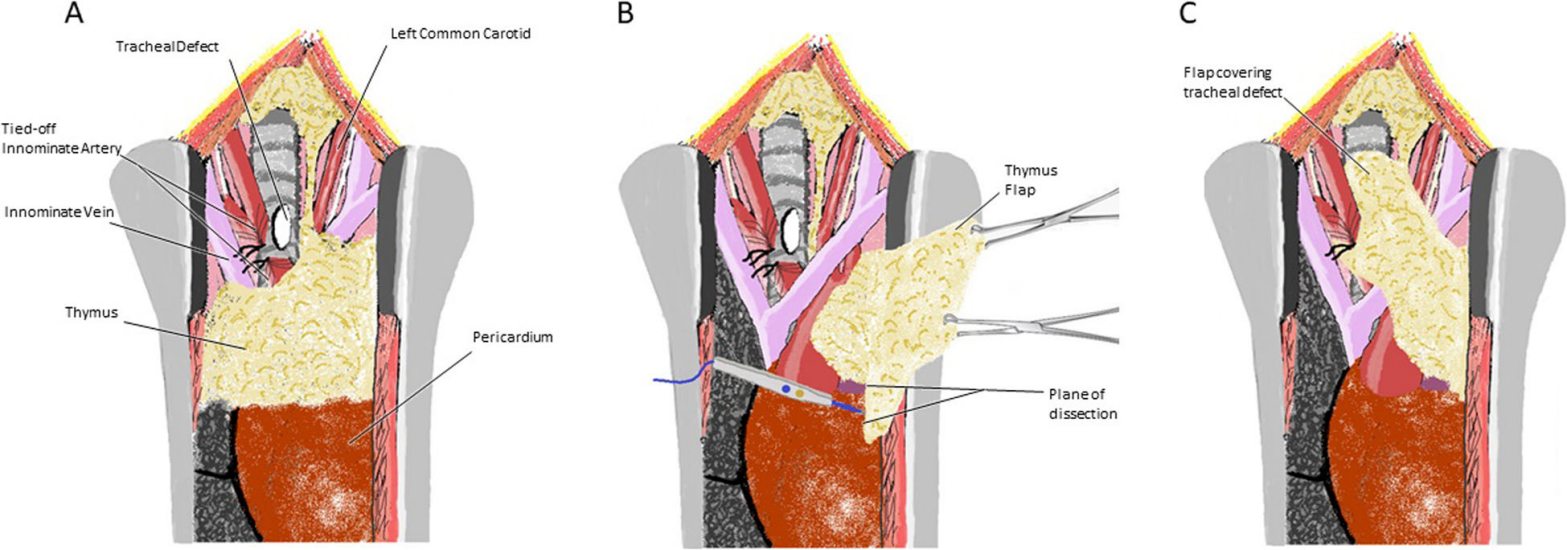
Pre-repair diagram (**a**), thymus flap mobilization (**b**), and tracheal repair (**c**)

The tracheostomy tube was then exchanged for an endotracheal tube, which was placed by anesthesia using a #8 anode tube under direct vision through the mouth and then directed down the stomal defect into the intrathoracic trachea. Intraoperative bronchoscopy was used to position this tube approximately 2 cm proximal to the carina. The cuff was inflated and was not projecting through the stomal defect. Due to significant contamination concerns of this large stomal defect, we proceeded with mobilization of the sternocleidomastoid muscles bilaterally with division of the omohyoids bilaterally. This was used to reapproximate the inferior portion of the defect using an interrupted 2-0 Vicryl suture in a figure-of-eight fashion. This left a large defect still present in the superior portion of the trachea. The patient was noted to have a rather large thymic fat pad, which was mobilized off the pericardial surface from the diaphragmatic insertion site. This was then flipped over at the level of the ascending aorta and dissected over to the level of the phrenic nerve on the left for complete mobilization. This was then placed with ease under no tension over the remaining portion of the tracheal defect and sutured to the skin with a 3-0 Vicryl suture, essentially obliterating the space (Figs. 2, 3, 4, and 5). The chest cavity was then irrigated with copious amounts of antibiotic solution followed by placement of vancomycin powder at the sternal edges. Chest tubes were inserted, and the wound was packed using povidone-iodine-soaked cling gauze rolls and temporary closure of the chest with an Esmarch covered with an Ioban dressing. The patient was then transferred to the critical care unit for further monitoring and ventilator management.

Following the procedure, the patient recovered from surgery and remained intubated. The patient did well initially; however, like many patients with tracheostomies for ventilator-dependent respiratory failure, he had many comorbidities. The patient developed recurrent *Clostridium difficile* colitis and he subsequently developed toxic megacolon. The patient’s family chose palliation rather than further surgery and he eventually succumbed to intra-abdominal catastrophe, unrelated to his tracheoinnominate artery fistula.

## Discussion

Patients with tracheostomies are at risk for the formation of a fistulous tract between the trachea and the innominate artery. The incidence of tracheoinnominate artery fistula formation is thought to be between 0.1 and 1% of patients with a tracheostomy in place [7]. The etiology of the tracheoinnominate artery fistula is considered to be multifactorial with factors ranging from pressure necrosis due to inflation of the cannula cuff, low tracheal incisions at the time of the index procedure, prolonged intubation, mucosal trauma from a malpositioned cannula tip, excessive neck extension or movement, and radiation therapy [2]. Additional factors include episodes of hypotension, corticosteroid use, malnutrition, and infection [3]. The fistula is often catastrophic because it carries an extremely poor prognosis. The majority of patients perish once the fistula forms. Even with immediate surgical intervention, more than 50% of patients do not survive, with another 56% of those who survive surgery expiring soon after surgery [1].

Surgical management of the tracheoinnominate artery fistula generally involves ligation of the tracheoinnominate artery, though some surgeons prefer to reconstruct the artery after resection of the fistula segment, since there is approximately a 10% risk of neurological sequelae, such as stroke, or other vascular complications in patients who have had ligation of the artery. However, it has been reported that there is also an increased risk of rebleeding in patients who have had reconstruction [6]. Other surgeons have advocated preventative ligation of the innominate artery in patients who are at high risk for developing a tracheoinnominate artery fistula despite the increased risk for stroke at the time of tracheostomy [6]. While there is currently no role for routine innominate artery flow assessment in patients who are status post a tracheostomy creation, Iodice et al. describe using computed axial tomography (CT) imaging to assess proximity of the innominate artery to the trachea in certain high-risk groups of patients. Those with impending tracheoinnominate artery fistula formation underwent epiaortic doppler and magnetic resonance arteriography to evaluate the presence of collateral circulation and to evaluate the risk of stroke in the setting of innominate artery ligation [6].

Bleeding in the operative field can greatly complicate life-saving intervention. For this reason, interventions which avoid the thorax can be appealing. Advances in endovascular surgery have provided another technique to manage a tracheoinnominate artery fistula. In certain select patients, an endovascular stent graft repair can be used as first-line treatment for management of the fistula and has been suggested as an option for bridging a patient with life-threatening bleeding until more formal surgical intervention can be performed [8].

Once the arterial component of the fistula is managed, either by resection or reconstruction, the tracheal defect must be repaired, especially in cases of arterial reconstruction. An autologous pericardial patch can be used [3]. In some cases, bioprosthetic materials may need to be used to close the defect in the trachea. Udelsman et al. reported successfully using an aortic homograft and acellular dermal matrix for closure of other types of tracheal fistulae (e.g., tracheoesophageal fistulae) [4]. Different vascular tissues such as a thoracic aortic patch, an abdominal aorta patch, and carotid patch with temporary stenting of the trachea have all been used successfully for repair of the tracheal defect. Microscopic analysis of biopsies taken from grafted tissue show metaplasia into well-differentiated tracheal tissue occurs after the trachea is repaired in this manner [5]. The authors reported absence of mucous secretions or peristalsis, less immunogenicity, and less vascularity when using a vascular patch.

Flap procedures are frequently employed for repair of the tracheal defect. The most common flaps include sternocleidomastoid muscle flaps, deltopectoral flaps with costal cartridge, pectoralis major muscle flaps with costal cartilage, and chondromucosal forearm flaps [5]. The use of a thymus flap, as was used in this case, has not previously been described in the setting of a tracheoinnominate artery fistula. The thymus has been used as a pedicled flap in repair of a tracheoesophageal fistula after primary repair for reinforcement, but after extensive literature review, only one published paper was identified in which the thymus had been utilized for closure of any tracheal defect, and this was for a controlled situation with a planned operation. In this case, a pedicled flap was created from the right lobe of the thymus and interposed into the tracheal defect [9]. The thymus in our patient was an ideal structure to use as a pedicled flap. It was in close proximity to the trachea and did not require significant bending or twisting to approximate it over the defect which could have placed its blood supply at risk.

Emerging technologies have provided alternatives for reconstruction of tracheal defects. Over the last 15 years, tissue-engineered tracheal (TET) patches have been developed which are formed from a decellularized porcine jejunal scaffold seeded with muscle and fibroblasts. Since 2004, there have been a number of studies that have shown that once implanted, they are covered with respiratory epithelium and are virtually indistinguishable from native respiratory tissue [5]. After the success of the jejunal scaffold, bioartificial materials have also been utilized, including 3D printed scaffolds that have been used to reconstruct large portions of the trachea. While these options have shown promise, they must be used in a pre-planned setting with extensive preoperative preparation.

Our patient eventually succumbed to toxic megacolon. Though it had nothing to do with the tracheoinnominate fistula, it deserves discussion. Toxic megacolon was first described in 1950 by Marschak et al. It occurs most commonly in the setting of *Clostridium difficile* colitis and ulcerative colitis and can progress to fulminant colitis which is almost invariably lethal if a colectomy is not performed [10].

## Conclusion

In patients with a tracheoinnominate artery fistula, the first priority for survival is to establish control of the hemorrhage. Following this, the trachea will require reconstruction. The use of a pedicled flap is often necessary for repair of this defect. We have shown that a pedicled flap of the thymus gland was effective for repair of a tracheal defect in the setting of a tracheoinnominate artery fistula.

## Abbreviations

AAA: Abdominal aortic aneurism
AF: Atrial fibrillation
CVA: Cerebral vascular accident
COPD: Chronic obstructive pulmonary disease
CT: Computed axial tomography
PEA: Pulseless electrical activity
ROSC: Return of spontaneous circulation
TET: Tissue-engineered tracheal [patches]
